# Identification of Potential Insect Growth Inhibitor against *Aedes aegypti*: A Bioinformatics Approach

**DOI:** 10.3390/ijms23158218

**Published:** 2022-07-26

**Authors:** Glauber V. Da Costa, Moysés F. A. Neto, Alicia K. P. Da Silva, Ester M. F. De Sá, Luanne C. F. Cancela, Jeanina S. Vega, Cássio M. Lobato, Juliana P. Zuliani, José M. Espejo-Román, Joaquín M. Campos, Franco H. A. Leite, Cleydson B. R. Santos

**Affiliations:** 1 Graduate Program in Network in Pharmaceutical Innovation, Federal University of Amapá, Macapá 68902-280, AP, Brazil; vilhenac@hotmail.com; 2 Laboratory of Modeling and Computational Chemistry, Department of Biological and Health Sciences, Federal University of Amapá, Macapá 68902-280, AP, Brazil; cassio153bio@gmail.com; 3 Laboratory of Biotechnology in Natural Products, Department of Biological and Health Sciences, Federal University of Amapá, Macapá 68902-280, AP, Brazil; aliciakarine1@gmail.com (A.K.P.D.S.); estersa07@gmail.com (E.M.F.D.S.); cancelaluanne@gmail.com (L.C.F.C.); js.vega@hotmail.com (J.S.V.); 4 Laboratory Molecular Modeling, State University of Feira de Santana, Feira de Santana 44036-900, BA, Brazil; moysesfagundes@gmail.com (M.F.A.N.); fhpharm@gmail.com (F.H.A.L.); 5 Laboratory Cellular Immunology Applied to Health, Oswaldo Cruz Foundation, FIOCRUZ Rondônia, Porto Velho 78912-000, RO, Brazil; juliana.zuliani@fiocruz.br; 6 Department of Pharmaceutical and Organic Chemistry, Faculty of Pharmacy, Institute of Biosanitary Research ibs, University of Granada, 18071 Granada, Spain; josemanuelespejo@correo.ugr.es (J.M.E.-R.); jmcampos@ugr.es (J.M.C.)

**Keywords:** *Aedes aegypti*, pharmacophore, molecular docking, insect growth inhibitor

## Abstract

*Aedes aegypti* is the main vector that transmits viral diseases such as dengue, hemorrhagic dengue, urban yellow fever, zika, and chikungunya. Worldwide, many cases of dengue have been reported in recent years, showing significant growth. The best way to manage diseases transmitted by *Aedes aegypti* is to control the vector with insecticides, which have already been shown to be toxic to humans; moreover, insects have developed resistance. Thus, the development of new insecticides is considered an emergency. One way to achieve this goal is to apply computational methods based on ligands and target information. In this study, sixteen compounds with acceptable insecticidal activities, with 100% larvicidal activity at low concentrations (2.0 to 0.001 mg·L^−1^), were selected from the literature. These compounds were used to build up and validate pharmacophore models. Pharmacophore model 6 (AUC = 0.78; BEDROC = 0.6) was used to filter 4793 compounds from the subset of lead-like compounds from the ZINC database; 4142 compounds (dG < 0 kcal/mol) were then aligned to the active site of the juvenile hormone receptor *Aedes aegypti* (PDB: 5V13), 2240 compounds (LE < −0.40 kcal/mol) were prioritized for molecular docking from the construction of a chitin deacetylase model of *Aedes aegypti* by the homology modeling of the *Bombyx mori* species (PDB: 5ZNT), which aligned 1959 compounds (dG < 0 kcal/mol), and 20 compounds (LE < −0.4 kcal/mol) were predicted for pharmacokinetic and toxicological prediction in silico (Preadmet, SwissADMET, and eMolTox programs). Finally, the theoretical routes of compounds **M01**, **M02**, **M03**, **M04**, and **M05** were proposed. Compounds **M01**–**M05** were selected, showing significant differences in pharmacokinetic and toxicological parameters in relation to positive controls and interaction with catalytic residues among key protein sites reported in the literature. For this reason, the molecules investigated here are dual inhibitors of the enzymes chitin synthase and juvenile hormonal protein from insects and humans, characterizing them as potential insecticides against the *Aedes aegypti* mosquito.

## 1. Introduction

Mosquitoes are one of the largest groups of insects (pests) with medical importance in the global control of disease transmission, and the highlight is the *Aedes aegypti* mosquito, the main transmitter of arboviruses such as dengue, dengue hemorrhagic fever, urban yellow fever, and chikungunya [1]. Until lately, the alarming growth of cases of these diseases in different regions of the world was restricted to emerging countries, now becoming a global concern. Currently, there is only one registered and widely distributed vaccine for yellow fever [2]. The way to reduce the spread of these viral pathologies is through the control of the transmitting mosquito; the main means is the use of chemical insecticides in the different stages of the mosquito’s development.

A class of these insecticides is called insect growth inhibitors (IGIs), which are used to control insect pests in agriculture, forestry, stored products, and public health situations [3]. IGIs act in the early stages of development (larval and pupal) and on two main neurohormones (neuropeptides), the ecdysteroids (molting hormones) and the juvenile sesquiterpenoid hormones (JHs), which have the function of controlling molting and metamorphosis [4].

However, in post-embryonic development, both hormones are related in adulthood to the regulation of reproductive maturation, release, transport, excretion, and metabolism as the main source for chitin synthesis and insect cuticle sclerotization [5,6,7]. A cuticle and attached epidermis are one of the largest and most important organs in the insect body, the function of which is to protect against physical damage, pathogen penetration, and desiccation. Associated with growth hormones, insects need to form new cuticles and periodically eliminate old ones; this involves many biochemical reactions, such as chitinization [8,9,10,11].

Chitin is a linear amino polysaccharide polymer composed of units of β-1,4-*N*-acetyl-d-glucosamine (GlcNAc). They are found in the cuticles of insects, crustaceans, and fungi and have the constitution of chains of α-chitin, considered the most abundant form of chitin since its form of organization is in a very stable antiparallel way. This form of sclerotization, depending on the levels of organization of the epidermis, creates structural and architectural differences where several cuticular proteins can be cross-linked with orthodiphenols and their quinones. In this way, the epidermis, composed of a single layer of underlying epidermal cells, is generated and can originate not only the differences within a species of insect but also among insects [12,13].

Eight enzymatic steps are necessary to convert the disaccharide precursor trehalose to chitin in the final step. The UDP-*N*-acetyl-glucosamine units are finally converted into chitin polymers by the enzyme chitin synthase, and this fact occurs at the limit of two topological spaces, from the cytosol in the lumen of the vesicles or outside the cell [10,13].

Therefore, there is great interest in insecticides that act specifically in the development of insects since their use on a large scale has provided resistance and also presented adverse reactions in other organisms, especially those with chitin (crustaceans). Therefore, through computational methods, it is possible to design compounds that meet the requirements of pesticides, as they are specifically developed for mosquito control.

**Scheme 1:**
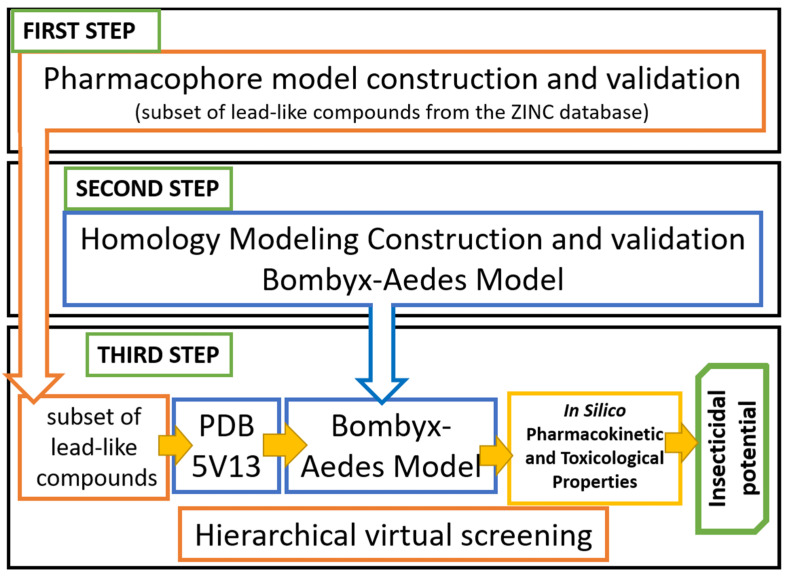
Methodological scheme. First stage (pharmacophore models) > second stage (comparative modeling) and third stage (hierarchical virtual screening—model and coupling).

The present work aims to search for new chemical entities with potential insecticidal activity with the dual inhibition of juvenile hormone protein and chitin deacetylases of *Aedes aegypti*, employing molecular modeling approaches by pharmacophore and docking-based virtual screening. In addition, compounds were developed through the pharmacokinetic characteristics (ADME) of *Aedes aegypti* physiology and toxicological properties directed at other organisms, mainly humans. The general outline that summarizes the methodological steps in this document is shown in Scheme 1 (see more details in the Materials and Methods section) [14].

## 2. Results and Discussion

### 2.1. Pharmacophore Model Generation and Evaluation

Ligand-based approaches can be useful in the prioritization of hit compounds with the same stereo-electronic features of known active inhibitors (e.g., pharmacophore models). Thus, the employment of potent compounds in pharmacophore model building is directly related to a reliable 3D representation of stereo-electronic features and pharmacophore quality [15,16,17].

First, all models presented strain energy of less than two orders of magnitude (<100 kcal/mol) and were all considered acceptable, complying with this condition. The non-dominant analysis provided by the Pareto index, the score of which is different from zero, would determine if there are dominant parameters that could affect the statistical quality of the models; this also did not occur [18]. Regarding the steric overlap for the model (Sterics), pharmacophore agreement (H_bond) and agreement between the characteristics of the compound used in the construction of the pharmacophore model in relation to the pharmacophore requirements (Mol_qry) revealed that they were statistically equivalent (Pareto = 0), and, therefore, this metric was not sufficient to select a useful pharmacophore model (see Table 1). In order to circumvent this limitation, the ability to differentiate actives compounds from false positives (decoys) was employed by the area under the ROC curve [15,19].

**Figure 1:**
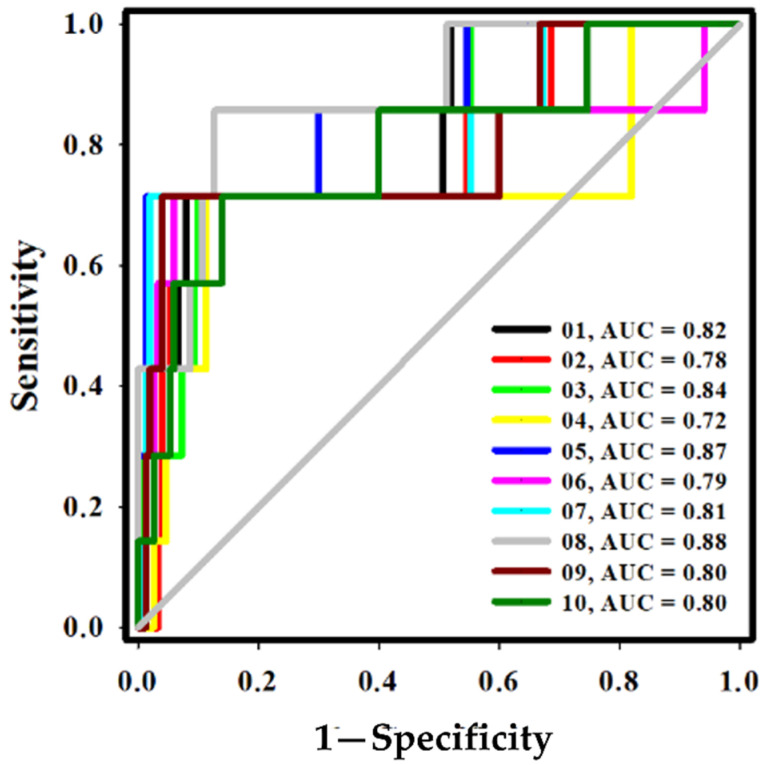
ROC curve for pharmacophore models. Below the diagonal line, poor models are represented for random selection (AUC < 0.5).

A test dataset with 7 inhibitors (chitin synthase—Ki < 0.001 mg·L^−1^ and juvenile hormone—Ki < 0.001 mg·L^−1^) and 350 decoys was employed in ROC curves built to calculate the respective areas under the curve (AUC-ROC) of each pharmacophore model (QFIT value; 0–100). An AUC equal to 1.0 would be found in a model with the capacity to recognize all actives before decoys, and an AUC less than 0.5 would be associated with models worse than a random selection. An AUC greater than 0.70 is associated with a moderate predictive ability [19]. According to the AUC calculation, all pharmacophore models had the ability to differentiate active compounds from false positives (AUC < 0.70; Figure 1).

Although all pharmacophore models had good predictive ability (AUC > 0.7), ROC curve analysis is a continuous metric, and thus, it is not able to classify active compounds in their early phases [20]. Thus, the Boltzmann-enhanced discrimination of the ROC curve (BEDROC) was employed to probe the pharmacophore models’ predictions to classify active compounds before decoys (Table 2).

Pharmacophore models with BEDROC > 0.5 are considered better than a random assay and useful for the early and ordered recovery of active compounds [21,22,23]. Although two pharmacophore models, models 06 and 09, showed satisfactory BEDROC values, pharmacophore model 6 was selected as it had the highest value for pharmacophore-based virtual screening.

Pharmacophore model 6, which was used in virtual screening, aims to find molecules that have in their structure the stereo-electronic requirements that are similar to the present model: two hydrophobic centers (HYs), two hydrogen bond donors (HBDs), and two hydrogen bond acceptors (HBAs). Compounds that have similarities to the physicochemical parameters of model 6 were identified since the program used was GALAHAD™ (Tripos Inc., St. Louis, MO, USA) for the alignment of very flexible compounds and different chemotypes. The adjustments were performed using a genetic flexible algorithm to align the compounds, allowing different conformations in the active site and defined by their conformers from the adjustments of the dihedral angles that were evaluated through a function of the scoring [15,24,25,26,27]. Structure–activity relationship studies for the most favorable recognition of macromolecules should consist of the aromatic presence, and the hydrophobic characteristics of hydrogen acceptance are an essential requirement for the recognition of chitin synthase [28,29]. For juvenile hormone protein, the donor and acceptor hydrogen bonds are essential parameters for binding to the protein [30,31].

**Figure 2:**
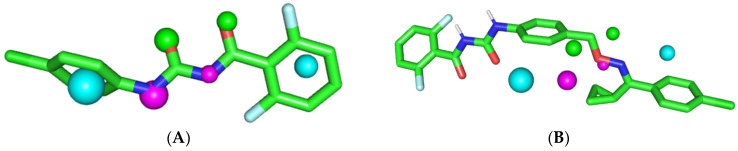
(**A**)—potent inhibitor (0.001 mg·L^−1^; QFIT = 84.94); (**B**)—flucycloxuron (0.05 mg·L^−1^; QFIT = 58.66), superposed to model pharmacophore 06. Spheres represent H-bond donors (green), H-bond acceptors (magenta) and hydrophobic groups (cyan). The size of spheres varies according to the tolerance radius calculated using GALAHAD^TM^. All the distances are measured in angstroms.

In order to characterize potent inhibitors with the pharmacophore model, a potent compound and a weak compound were superimposed on pharmacophore model 6. Compounds were analyzed experimentally by Sun et al. [32], and the compound with a value of 0.001 mg·L^−1^ was considered potent; the weak compound was the insect growth inhibitor flucycloxuron, used as a positive control (0.05 mg·L^−1^) (Figure 2).

The objective of the screening was to obtain a compound that has a dual action on both chitin synthase and juvenile hormone receptors. Pharmacophore model 6 allowed us to reproduce the requirements for chitin synthase and the recognition of the juvenile hormone receptor; therefore, it is useful to prioritize the potential inhibitors of *Aedes aegypti* insect growth regulators.

### 2.2. Pharmacophore-Based Virtual Screening

In total, 214,446 compounds were prepared in multiple protonation states and multiple tautomeric forms and were collected in structure data format (SDF), which is available in multiple conformations from the Sigma-AldrichTM subset of the ZINC [33,34] database (https://zinc15.docking.org/, accessed on 12 June 2021). The database of previously prepared compounds will be able to predict compounds with better properties, such as lipophilicity and water solubility [35,36]. For each molecule in the database, up to 300 conformers were generated using the MMFF94 force field implemented with OMEGA software (Open Eye Scientific Software, Santa Fe, NM, USA, http://www.eyesopen.com, accessed on 12 June 2021) [37], and the partial atomic charges were calculated by means of the empirical Gasteiger-Hückel method. Compounds that comply with the previous conditions are better suited for molecular docking [38]. Then, 99,623 compounds overlapped pharmacopeia model 06 with partial stereo requirements (0.47 < QFIT > 98.12), with the QFIT score being considered as a parameter. A score of 100 demonstrates that a better fit between the pharmacophore model and the database has occurred; when this score is zero, no overlap has occurred [39]. As a pharmacophore-based virtual screening result, 4793 compounds (QFIT > 58.94) were prioritized with QFIT between 58.95 and 98.12 [23,39,40]. However, this approach is not useful for evaluating the spatial requirements of the binding site. Thus, molecular docking was applied to the filtered compounds from the pharmacophore-based virtual screening.

### 2.3. Construction of the Bombyx-Aedes 3D Model and Molecular Docking

The macromolecule deposited in the PDB-encoded 5ZNT is a chitin deacetylase 1 protein. Although the CDA1 gene of the *Bombyx mori* organism, without a mutation, has no crystallized ligand, Liu [41] determined the structural and biochemical characteristics of the catalytic mechanism experimentally since the enzyme is classified as a hydrolase; its catalytic activity has been recorded (EC: 3.5.1.41): [(1 → 4)-*N*-acetyl-β-d-glucosaminyl] (n) + n H_2_O = n acetate + chitosan [42].

The primary structure of the *Aedes aegypti* species was searched with the descriptives (*Aedes aegypti* + chitin deacetylase 1 protein) at https://www.ncbi.nlm.nih.gov/protein, accessed on 27 June 2021, and we found that the protein AAEL003419-PA with 535 amino acids was used for the alignment through BLAST. From similar sequences found, the most compatible organisms (with the highest percentage of identification, above 80%, and consultation coverage above 70%) were selected [43].

**Figure 3:**
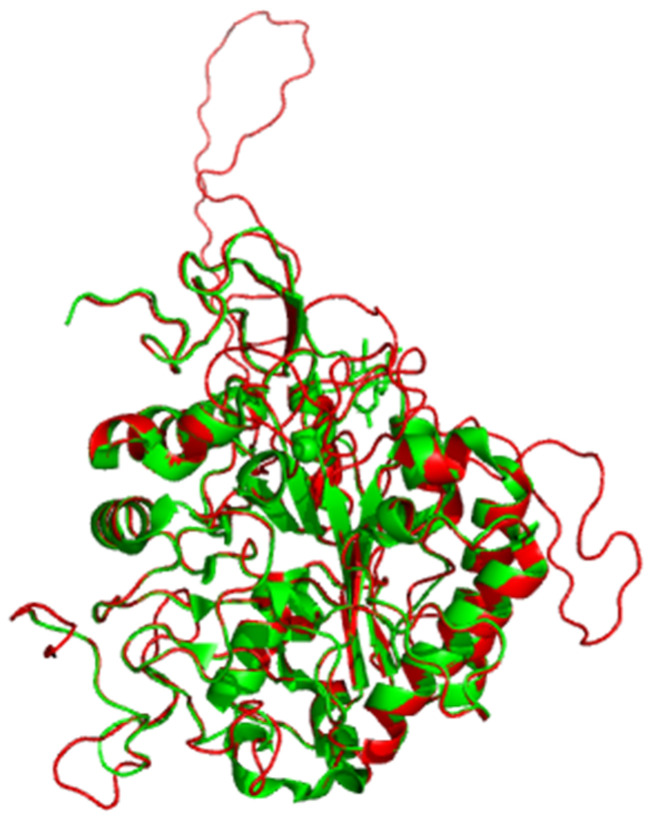
Overlap between alpha-carbons of macromolecule 5ZNT (green) and the Bombyx-Aedes model (red); RMSD value 0.396.

In the following steps, built in the MODELLER 10.1 program, the comparison, alignment, and construction of the Bombyx-Aedes model and its validation were performed. The model generated by the program was aligned in the Pymol 2.0.7 program (Warren Lyford DeLano, The PyMOL Molecular Graphics System, Schrödinger, LLC, New York, NY, USA) as shown in Figure 3, in which the RMSD alignment overlap was 0.396, from which the choice of the best atomic model with a lower score is considered adequate in its structural composition and reliability according to Rahman et al. [44].

**Figure 4:**
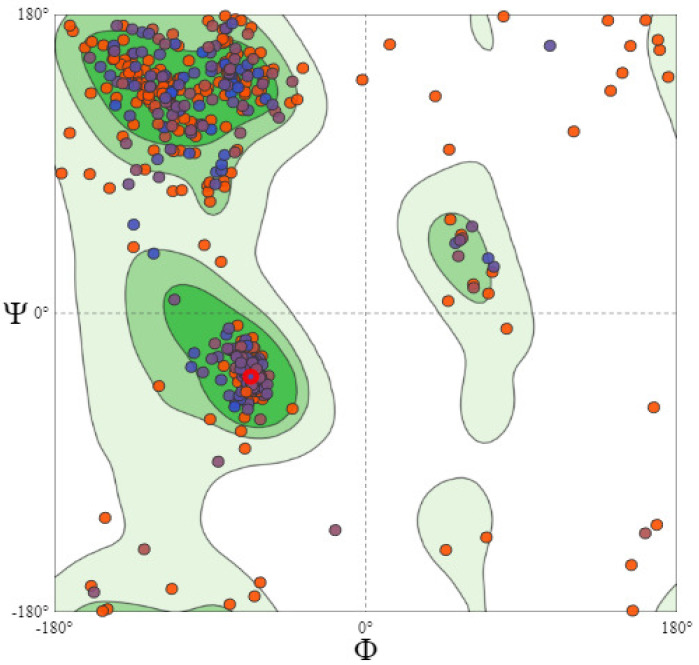
Ramachandran, the Bombyx-Aedes Model terrain. The most favorable regions are shown in dark green, neutral in light green, and non-favorable in white; each amino acid residue is marked with different colors, generated by the structure assessment program (https://swissmodel.expasy.org/assess/KGJVtX/01, accessed on 27 June 2021).

The Bombyx-Aedes model presented a percentage of more than 90% of residues in favorable regions, demonstrating a good stereochemical quality, as analyzed by the Ramachandram plot and shown in Figure 4. The Ramachandran plot of the Bombyx-Aedes model shows the distribution of the torsion angles Φ and Ψ of the main chain of the model’s built protein [45]. The Ramachandram plot is divided into different zones: (a) those where the color intensity is least intense because of the fact that there are no interatomic clashes; (b) those where there are moderate clashes, and (c) those where the clashes are extremely severe, with the most intense coloration [46]. A Global Model Quality Estimate GMQE value of 0.5 was found, indicating a model with higher reliability.

Carbohydrate esterases of family 4 (family CE-4) are metalloenzymes; predominantly, they use Co^2+^ and Zn^2+^ ions as divalent metal cofactors in vitro. These enzymes are constituted by a structure formed by a triad of zinc and His–His–Asp bonds, acting as a catalytic domain and an acid and base. It has two oxygenated acetates that coordinate zinc in a distorted octahedral shape [42,47,48,49]. According to Dong [50], a recombinant form of peptidoglycan GlcNAc-*N*-acetylase from *S. pneumoniae* (PgdA_Sm_) binds to a zinc ion within its active site and in vivo and demonstrates that the protein PgdA*_Sm_* can be fully activated by the presence of the zinc ion. Since any modifications to the CE4 motifs can provide a means to change the specificity of the substrate, a preference for the metal or its reaction mechanism represents a step towards the identification of mechanism-based inhibitors for this important class of enzymes [51].

#### 2.3.1. Selection, Validation, and Virtual Screening for Potential Dual Inhibitors 

The molecular adjustment was performed using the AutoDock Vina program, and all parameter values were adjusted throughout the process to define receptors, exhaustiveness, center_xyz, and size_xyz. To generate the result in binding affinity values, the AutoDock Vina program foresees the connection of the lowest energy state at the time of the formation of the protein complex (receptor) with the ligand, with the objective of a correlation between the values of the functions and the affinity of the ligand [52,53,54,55]. Among the 4793 compounds prioritized in the virtual pharmacophore-based screening of the Zinc15 database, 4142 compounds (ΔG < 0 kcal/mol) were aligned to the binding site of the crystalline structure of the juvenile hormone protein macromolecules (code PDB: 5V13) of *Aedes aegypti*; its complexed binding site has the reference ligand JH3 (methyl (2*E*,6*E*)-9-[(2*R*)-3,3-dimethyloxiran-2-yl]-3,7-dimethylnona-2,6-dienoate). Therefore, 2240 (LE <−0.40 kcal/mol) compounds were chosen since the efficiency of the ligand selects a more concordant compound for the choice of a principal compound [56,57,58].

**Figure 5:**
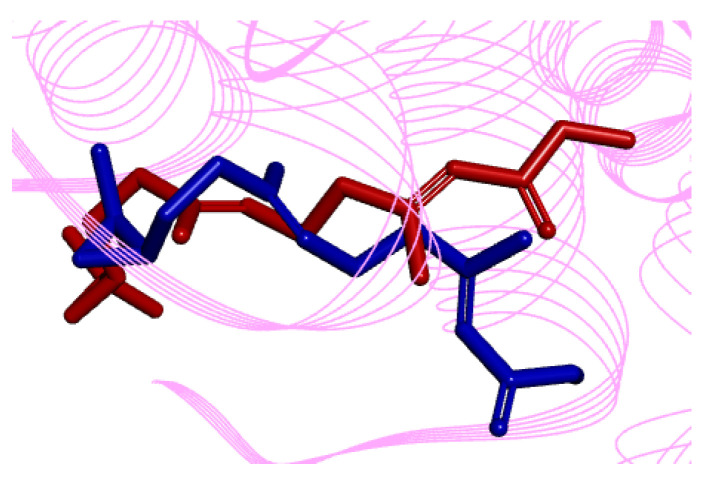
Overlays of crystallographic ligand poses in **JH3** (in red) with the calculated pose (in blue).

Molecular docking was performed using a validation protocol to determine the pose (conformation + orientation) of the ligands (**JH3**) within the enzyme active sites (PDB ID 5V13). The root mean square deviation (RMSD) between the reference ligand and the experimental **JH3** ligand (1.19 Å) was calculated. The RMSD analysis was determined by considering the most appropriate position of the initial structure around the X-ray crystallographic complex through an experiment of the most stable position so that there is no structural change between the protein and the reference ligand when complexed with the macromolecule under study. The best molecular fit is determined by an RMSD less than or equal to 1.5 [59,60,61,62,63], as shown in Figure 5.

Kenny et al. [64] stated that it does not seem possible to quantify the absolute binding efficiency for compounds objectively. The efficiency can still be defined in a relative way by scaling the affinity differences by the corresponding molecular size differences, and the differences were prioritized for molecular docking for the chitin deacetylase model *A. aegypti*.

Molecular docking to the binding site of the crystalline structure of the macromolecules of the juvenile hormone protein (PDB code: 5V13) was carried out on all compounds, and, after filtering the ligand efficiently, 2240 compounds went on to the next stage of molecular docking with the model Bombyx-Aedes enzyme insect chitin deacetylase built from the *Aedes aegypti* mosquito.

#### 2.3.2. Interaction Analysis of Inhibitors and Juvenile Hormone (PDB 5V13)

The juvenile hormone protein presents in its binding site the main interaction with the epoxy group and forms a hydrogen bond with the phenolic hydroxy group of Tyr129; the rest of the isoprenoid chain is surrounded by hydrophobic side chains, including those of Phe144, Tyr64, Trp53, Val65, Val68, Leu72, Leu74, Val51, and Tyr33 [37,65].

**Figure 6:**
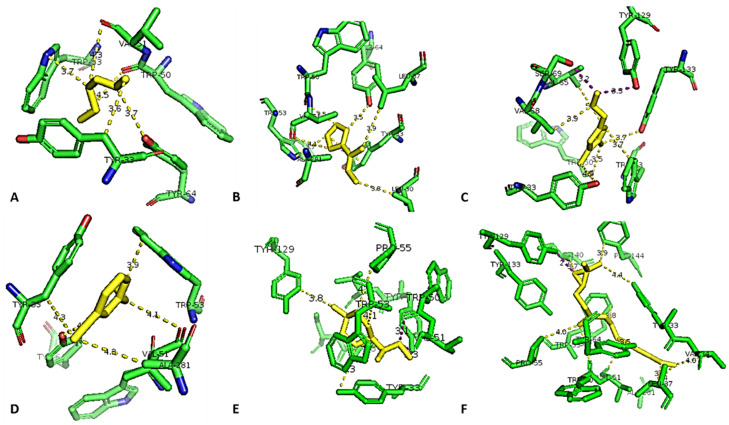
Interaction of selected molecules, in yellow, with the amino acid residues, in green, of the macromolecule 5V13 (juvenile hormone), in green. (**A**) **M01** (ZINC13514543); (**B**) **M02** (ZINC14413017); (**C**) **M03** (ZINC05680928); (**D**) **M04** (ZINC04691148); (**E**) **M05** (ZINC95851150); (**F**) **JH3**.

When we analyzed our ligands, important interactions were carried out with the main amino acid residues of the binding site of the *Aedes aegypti* juvenile hormone macromolecule (COD: 5V13). All selected ligands (**M01**–**M05**) and the reference ligand complexed with the macromolecule (**JH3**) interacted with residues Tyr64 (except **M03**), Trp53, Trp50 (except **M04**), Try33 (except **M03** and **M05**), and Val51 (except **M03** and **M04**). **M03** was the only one that interacted with the amino acid residues Val65 and Val68, as shown in Figure 6.

#### 2.3.3. Interaction Analysis of Inhibitors and Enzyme Model Insect Chitin Deacetylase from *Aedes aegypti*

The selection of compounds took place in a double inhibition of macromolecules, where the parameter to which they were associated performed more satisfactorily in filtering the choice of the most appropriate ligand that would, in particular, function as both an inhibitor of the juvenile hormone and a growth regulator of the *Aedes aegypti* mosquito. In view of this approach, 2240 (LE < −0.40 kcal/mol) compounds were selected in the first stage with the juvenile hormone macromolecule. For the next stage, there was, again, a selection of the molecular docking based on the insect model *Aedes aegypti* chitin deacetylase, constructed from the enzyme insect chitin deacetylase deposited in the PDB (PDB code: 5ZNT); 1959 compounds (ΔG < 0 kcal/mol) were aligned and 20 compounds (LE < −0.4 kcal/mol) were prioritized for pharmacokinetic and toxicological prediction.

**Figure 7:**
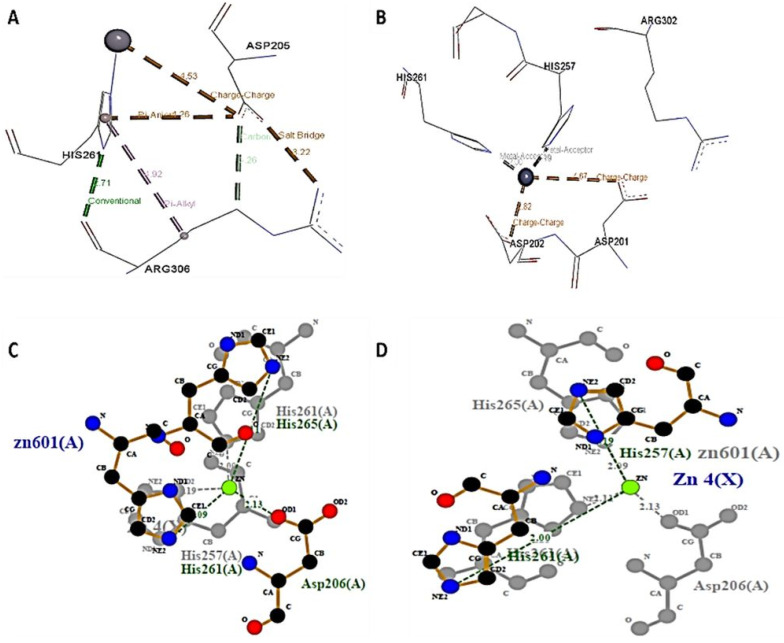
Interactions in the catalytic region around the metal zinc in the 5ZNT protein (**A**) and the Bombyx-Aedes model (**B**) and the overlapping of the amino acid residues closest to the zinc metal in the 5ZNT protein (**C**) and the Bombyx-Aedes model (**D**).

When we analyzed the interactions described by Liu [41], the 5ZNT protein, with the presence of the amino acid residues observed experimentally (Asp205, His 261, and Arg306; Figure 7A), and the interactions in the Bombyx-Aedes model (Figure 7B; Asp201 and 202, His257 and 262 and Arg302) maintained the same regions around zinc. This demonstrates the conservation of important amino acids for carrying out catalytic activity and, in Figure 7C, the overlap of the 5ZNT protein with the Bombyx-Aedes model, with the presence of amino acid residues closer to the metal (Asp206, His261, and 265). Figure 7D shows the overlapping of the Bombyx-Aedes model protein with the 5ZNT protein, with the presence of amino acid residues closer to the metal (His257 and 261).

In addition, the chitin deacetylase enzyme catalyzes the removal of acetyl groups from chitin and modifies this polymer during its synthesis and reorganization. The enzyme has a structure of two regions: a subdomain of NodB homology conserved in CE4 and C-terminal loops. The homology domain of NodB is a barrel (β/α)_7_ (residues 162–471) composed of seven parallel β strips arranged in a barrel surrounded by six α helices. The active site and the substrate connection gap are located through the upper center of the barrel (β/α)_7_, which contains a metal bond triad conserved throughout the CE4 family, namely, a zinc ion coordinated by Asp206 and Asp205, His261 and His265, in addition to Arg306 and canonical Arg306, responsible for stabilizing the catalytic base Asp205 [41,42].

A long crack in the substrate was also observed on the surface of the insect chitin deacetylases (CDAs) from the *silkmoth Bombyx mori* (BmCDA1-CAD), with only one hydrophobic residue exposed to the solvent (Tyr242) [41] that can help in the bonding of the substrate. From these interactions, the program is able to score the best connection with the most promising compounds.

**Figure 8:**
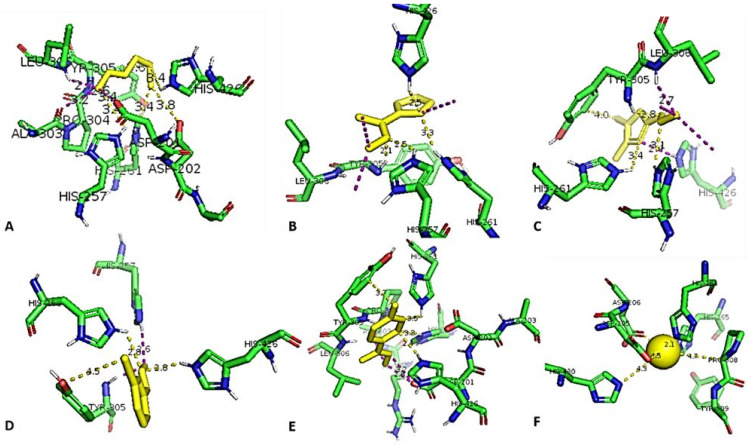
Interaction between the selected molecules, in yellow, with the amino acid residues, in green, of the macromolecule Bombyx-Aedes model, in green. (**A**) **M01** (ZINC13514543); (**B**) **M02** (ZINC14413017); (**C**) **M03** (ZINC05680928); (**D**) **M04** (ZINC04691148); (**E**) **M05** (ZINC95851150); (**F**) Zinc Ray.

When analyzing the ligands with the best scores, interactions of all ligands (**M01**–**M05**) and zinc with the amino acid residues His256, His261 (except M01), and Tyr305 (except zinc) were observed. Only compounds **M01** and **M05** interacted with the amino acid residue Asp302, and compounds **M01** and **M03** were the only ones that interacted with His430 and Leu306. Compounds **M01**, **M04,** and **M05** interacted with Ala303, as shown in Figure 8.

After analysis, 20 compounds with the best score values were selected for pharmaco-kinetic and toxicological screening in search of potential insecticide growth regulators. However, this approach is not useful for evaluating the permeability of compounds via the biological barriers of mosquitoes and the toxicological potential in humans. Thus, in silico pharmacokinetic and toxicological prediction was applied to the filtered compounds from the docking-based virtual screening.

### 2.4. In Silico Pharmacokinetic and Toxicological Properties

The development of a potential insecticide that can exercise its possible mechanisms of action by inhibiting the growth of insects must have a concentration that is sufficient to remain bioactive with its effective action. For this to occur, the insecticide needs to cross various barriers, such as the insect’s cuticle or trachea [37], as demonstrated by commercial insecticides. Their possible mechanisms of action include the inhibition of the enzyme chitin synthase located in the cuticle or the juvenile hormone protein or the insect ecdysone (diflubenzuron and buprofezin) located in the insect’s central nervous system (CNS).

Accepting that the insect’s physiology is a key point in the development of a new insecticide, the in silico pharmacokinetic properties that have been developed mainly include properties inherent to humans. However, the planning of an insecticide is based on some properties that are shared by other live organisms since the physiology is similar in some physicochemical properties [66,67,68,69]. In this study, four positive controls (diflubenzuron, DFB, teflubenzuron, TEF, flucycloxuron, FCX, and buprofezin, BUP) were used. These are insecticides that are on the market and have a proven action in inhibiting the growth of insects; they are used as references in relation to physical and chemical properties.

Costa et al. [37] described the possible ways of better penetration and action of an insecticide to gain access to the central nervous system at the adult stage of the insect, which is through the cuticle, trachea, and integument. Simon-Delson [70] and Denecker et al. [71] stated that it is common for insecticides to penetrate through the route of ingestion by the insect. However, another way is through its endocuticle in the larval period, when this tissue is in the development phase, with a large production of chitin synthase and growth hormones [72,73,74,75].

However, depending on the route that the insecticide takes, the insect’s physiological system will have several means to reduce the effect of an insecticide: for example, low penetration of the insecticide, where it does not reach sufficient concentration to exert its effective action, and where the insect has a high excretion of several insecticides, characteristic of its resistance to xenobiotics, as reported in the literature [1,76].

The planning of the insecticidal potential was developed with the screening of 20 compounds from the previous steps, of which five compounds were chosen; they had a high power of penetration through the insect pathways and sufficient quantity to inhibit the enzyme chitin synthase or the protein of the juvenile hormone or ecdysteroid hormone [77], which are the possible target receptors. 

Figure S1 (supplementary material) shows the positive controls and compounds that possibly have these properties, as can be seen on the bioavailability radar generated by the SwissADME program, in which the pink area represents the ideal range for each property (lipophilicity: XLOGP3 between −0.7 and + 5.0, size: MW between 150 and 500 g/mol, polarity: TPSA between 20 and 130 Å^2^, solubility: log *S* not exceeding 6, saturation: sp^3^ hybridization carbon fraction >0.25, and flexibility: <9 rotating links) [78,79].

We checked the radar bioavailability graph, which has six axes of important bioavailability properties for the absorption of the insect’s body wall and CNS. Of the positive controls DFB, TEF, FCX, and BUP and compounds **M01**–**M05**, as seen in Figure S1 (supplementary material) with possible insecticidal activity, BPU and **M03** were the only ones that did not violate any of the parameters, the pharmacokinetic properties of which remained within the range of the graph. The positive control FCX, on the other hand, violated the polarity parameter, making it a very bad insecticide to achieve high concentrations in the target receptors.

However, DFB, TEF (positive controls), **M01**, **M02**, **M04,** and **M05,** as per Figure S1 (supplementary material), violated the saturation property with an sp^3^ hybridization fraction greater than 0.25, demonstrating that the design of the compound substituents could increase the complementarity of receptors/ligands, making it possible that additional protein–ligand interactions would not be accessible to a flat aromatic ring and, therefore, would improve potency and selectivity for a given target, which should mitigate the off-target effects [80].

**Figure 9:**
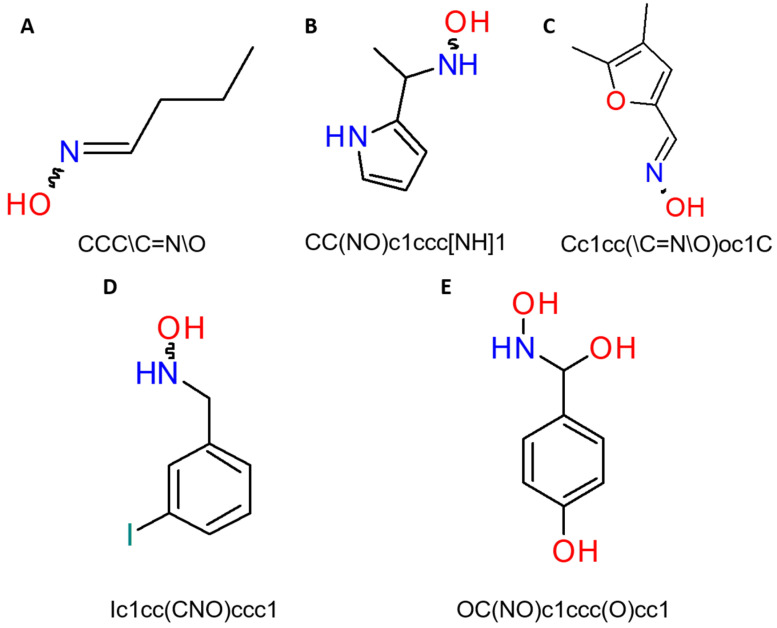
Promising compounds (**A**) **M01** (ZINC13514543); (**B**) **M02** (ZINC14413017); (**C**) **M03** (ZINC05680928); (**D**) **M04** (ZINC04691148); (**E**) **M05** (ZINC95851150). Source: authors.

It is clear from this data that a greater saturation would allow higher complexity of the compounds, including the presence of stereocenters. Such compounds will have access to more available chemical space, making possible the complementarity of the spatial connections of the active sites of the target protein [80], criteria obeyed by **M01**, **M02**, **M04**, and **M05** (Figure 9). However, saturation will also affect physical properties: saturation has been correlated with solubility; the more saturated the compound, the greater the likelihood of solubility [80].

Thus, as the increase in log P_o/w_ leads to a decrease in aqueous solubility, analyzing another method of solubility that influences the capture, distribution, transport, and, eventually, the bioactivity of a drug at the site of its actions could facilitate drug development, mainly in the ease of handling and formulation [81,82]. The FCX compound again differs in value, as expected, and cannot cross the blood–brain barrier easily. According to Table 3, the positive controls DFB and TEF are poorly soluble, whereas BUP and all planar compounds are soluble in an aqueous system. To ratify our results regarding the penetration of the blood–brain barrier and absorption by the insect cuticle, we used the data presented by the Preadmet tool, which makes biological predictions that show the same relationship of the compound’s passage to reach the studied enzymes in sufficient concentrations to exercise its effect. The TEF and FCX compounds did not cross the blood–brain barrier due to their log P_o/w_ and *S* results (see Table 3).

Therefore, according to the literature, the greater the hydrophobic property, the greater the log*P* value. The parameters were analyzed to determine whether the insecticide can overcome the cuticle and blood–brain barrier. Another parameter of biological activity that is considered a good model for analyzing the blood–brain barrier is the apparent cell permeability property, in nm/s, of two renal cell lines from Madin-Darby dogs (MDCK) [83,84,85,86,87], as can be seen in Table 3, following our virtual screening based on hieratic ligands, where we selected, according to the criteria above, five compounds in the data set that had values within the parameters described by the literature.

The pharmacokinetic properties are directly related to the proper planning of the insecticidal potential since different substituents on a given chemical entity are used as references to potentially create more efficient compounds according to their hydrophobic, steric, and electronic characteristics. Therefore, when analyzing our compounds, we take into account the octanol–water partition coefficient to define the hydrophobic character of a substituent [88] on different platforms that are condensed by the SwissADME program [79].

The SwissADME program for better visualization allows the BOILED-Egg method of estimated brain or instinct permeation, an intuitive method to simultaneously predict two main parameters of the ADME, that is, passages across membranes and brain access (BBB). Although conceptually very simple, as it has only two physical–chemical descriptors (WLOGP and TPSA, for lipophilicity and apparent polarity), this classification model was constructed with extreme care for statistical significance and robustness [78,79,89].

The selection of compounds **M01**–**M05** was based on the structural simplicity of the derivatives and the fact that it allows the study of their effects on the cuticle and CNS. Since solubility is one of the main physicochemical properties for the development of a drug, the partition coefficient between *n*-octanol and water in its logarithmic form (log P_o/w_) could determine the assessment of the pharmacological properties of absorption, distribution, metabolism, excretion, and toxicity (ADMET) of a candidate chemical lead for its initial selection. It establishes benchmarks against which compounds synthesized during lead optimization can be evaluated [90,91].

Log P_o/w_, processed by the SwissADME program, as can be seen in Table 3, uses five multiple computational methods (iLOGP, XLOGP3, WLOGP, MLOGP, SILICOS-IT) for estimating and increasing the accuracy predictors of this physical–chemical property and generating the consensus log P_o/w_. This parameter is the arithmetic means of the values predicted by the five proposed methods based on free solvation energies in *n*-octanol and water, calculated by the generalized-born model, and can give an estimation of the solvent-accessible surface (GB/SA) [92,93,94,95,96]. As to the parameters established in the literature and by positive controls, only the FCX control presented a value above 5 (consensus log P_o/w_ of 5.37); however, this compound can overcome the gastrointestinal barrier, which in the insect’s structure would be the passage through the cuticle. However, it cannot overcome the blood–brain barrier in sufficient concentrations due to its atomic nature, with three aromatic rings, together with a cyclopropane, making it possible to increase the log P_o/w_ [97,98,99].

From the result of Table 3, four compounds that allowed the best entry into the absorption of the central nervous system (CNS) through the blood–brain barrier and one that had excellent absorption properties through the membrane were selected, as shown in the graph (see Figure S2 in the supplementary material), in which the white and yellow regions (yolk) are equivalent to the area most likely to be absorbed by the cell and most likely to access the brain (penetration), respectively. In agreement with Geldenhuys [90], who, in his study, developed a computational approach for the brain uptake of compounds in an insect model system, we concluded that in silico database filtering models of small compounds used in drug discovery were able to express the correlation between the BBB models. When analyzing the graph of the three growth-inhibiting insecticides on the market, DFB and BPU, together with compounds **M01**, **M02**, **M03**, and **M04,** are found in the yellow region (yolk) [89]. On the other hand, the insecticides TEF and FCX, and compound **M05** are in the white region of HIA (gastrointestinal absorption), corroborating previous data. When performing the in silico experiment in the SwissADMET program for the 20 compounds, the program did not generate results for compounds ZINC95851157 and ZINC12447446. They cannot be seen in the graph because their results are out of reach, which can be explained by the fact that they have deficient pharmacokinetic properties, similar to the BCR-ABL-1 inhibitor. They are predicted with an accuracy of 83% (true negatives are out of range) [78].

This is a clear indication that these compounds are highly likely to be absorbed by the gastrointestinal tract and permeate the insect’s brain; the compounds TEF, FCX, and **M05** did not cross the blood–brain barrier in a high concentration, in spite of our plan for the growth inhibitors’ possible mechanism by inhibiting the enzyme chitin synthase present in the cuticle. Another analysis parameter in the graph is the possibility of active efflux from the cell to expel the compound that passes through the barriers that are substrates or non-substrates of the permeability glycoproteins PGP^+^ (substrates) and PGP^−^ (not substrates). Blue and red dots symbolize compounds; none of the compounds were PGP^+^, shown as blue and red dots on the graph, and none of the compounds were PGP^+^. Probably they prevent the intracellular accumulation of toxic compounds, as they are eliminated outside the cell through active efflux through biological membranes [91,92,93].

The glycoprotein-P of the *Aedes aegypti* mosquito shows similarity in its identity to other species of insects and to humans [94]. It was observed in the literature that the baseline ATPase activity of ATP-binding cassette transporters in larvae increased insecticide efflux in the presence of organophosphate larvicides (temephos); a growth inhibitor (diflubenzuron) showed a significant increase in the toxicity of these insecticides [95]. However, Figure S2 (supplementary material) shows that all positive controls and the studied compounds were PGP^−^ since this depends on the species of insects, the structural components of the compounds, and how they are analyzed [94,96,97,98]. 

To design an insecticide that is developed based on certain mechanisms of action, be it the chitin synthase enzyme or the *Aedes aegytpi* juvenile hormone protein, it is expected that the insecticide requires a minimum concentration for the desired effect. A toxicity assessment in humans is essential for any insecticide [99]. Shown in Table 4, toxicological predictions were made using the eMolTox server (http://xundrug.cn/moltox, accessed on 30 June 2021).

EMolTox is a web server for predicting potential toxicity associated with the structure of a compound; it predicts the compound with high confidence using the aggregate predictor method (ACP). It was elaborated based on literature data from a total of 174 in vitro/in vivo experimental data sets related to toxicology. The data were used to build the model through physical–chemical descriptors and Mondrian’s shaped prediction, which offers similar prediction accuracy for both active and inactive sets, where the validity values are around 0.90, at a significance level of 0.1. This toxicological web server predicts the molecular toxicity of the target molecule based on the mechanism of action in different animal models and their organs [100,101,102,103,104,105,106,107,108,109,110].

The toxicological web server eMolTox presented the following data, of which the potential injury alerts for genotoxicity were for the positive controls FCX and BUP, in addition to injury to the liver and endocrine, whose actions are in some pathway signaling that is similar to foreheads in vitro. As for the DFB and TEF positive controls, the alerts for injuries were in the liver, nervous system, immune system, and kidney, and their actions were in modulators; all confidence levels were higher than 0.981. On the other hand, only the **M04** compound was on alert for skin injury, together with alerts in the liver for the action of blocking the (OATP1) B1 transporter, which is the same for the **M02** and **M03** compounds. The organic anion transporter polypeptide (OATP) 1B is a detoxifying limiting protein that mediates the uptake and elimination of many endogenous and xenobiotic compounds (drugs or insecticides) through the sinusoidal membrane of liver cells [111,112,113,114,115]; **M02**, **M03**, and **M04** are possibly blocking this transporter, making them molecular structures to be eliminated by living beings. **M01** and **M05** did not show any actions or injuries (Table 4).

The possible toxicity of insect growth inhibitors specific to several species has been analyzed in the literature. It can be found in the Scientific Opinion of the Panel on Plant Protection Products and Their Residues (PPR Panel), at the request of the PRASA Unit of the European Food Safety Authority (EFSA), on the genotoxic and carcinogenic potential of buprofezin in the context of human risk assessment. It was evaluated in a two-year study that, in mice and rats, neither the nature nor the incidence of tumors was affected by its administration [116]. However, with teflubenzuron, the main target organ of which is the liver, after short or long-term exposure in species of rats, mice, or dogs, hepatotoxicity was observed in an 18-month oncogenicity study in mice. Benign hepatocellular adenomas, observed only in this study with mice, were not considered relevant to human exposure [117].

In one study, BUP and organophosphates were tested for chromosomal aberrations in somatic and germ cells in mice, induced by high doses. The growth inhibitor was a relatively safer compound than conventional organophosphate insecticides [118]. It is undoubtedly common that the toxicity of BUPs is related to their metabolites because they are degraded by hydrolysis and photodegradation, generating metabolites 2,6-difluorobenzamide, 4-chlorophenylurea, 4-chloroacetanilide, 4-chloroaniline, and *N*-methyl-4-chloroaniline, of which the last three are classified as mutagenic [119,120]. It was observed in blue-green algae (*Plectonema boryanum*) that when the phenylurea derivatives were exposed and became highly resistant, the main metabolite was *p*-chlorophenylurea [1,119,121]. None of our **M01**–**M05** compounds (Figure 9) were planned without the chlorine atom, and, therefore, they have promising insecticide potential without the mutagenic effect.

The compound that showed excellent results for the standards established by the program when analyzing Table 4 was compound **M05**, which did not present any toxicity. Regarding hepatoxicity [122,123] and cytotoxicity, all positive controls were alerted as active, while **M02** and **M03** compounds were alerted as a transportation block in the liver; together with **M04** (alerted for skin sensitivity), these compounds could give rise to a few adverse reactions. Teflubenzuron (TEF) is used in the delousing of salmon farms; analyses in non-target species such as lobster (*Homarus gammarus*) and shrimp (*Palaemon elegans*) have shown exoskeletal abnormalities (deformities of the claws or rigid legs); deformities of juvenile lobsters have been observed in several organs [124,125,126].

### 2.5. Structure–Activity Relationship of the Promising Molecule

**Figure 10:**
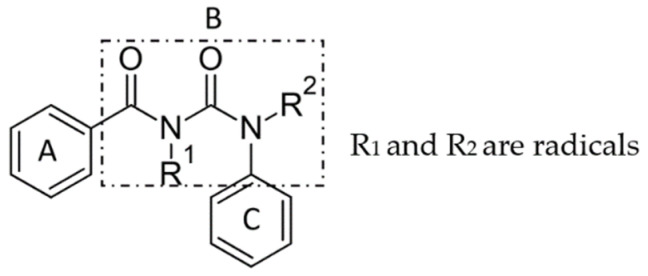
The basic chemical structure of benzoylphenylurean compounds consists of three regions for SAR analysis: the benzoyl ring (A), the urea bridge (B), and the aniline (C).

Insect growth inhibitors possess the basic chemical structure of benzoylphenylurean compounds, which consist of three regions for SAR analysis: the benzoyl ring (A), the urea bridge (B), and the aniline (C), as shown in Figure 10. The best-known insecticide is diflunzuron, which has a structure composed of benzoyl urea, similar to sulfonylureas used as oral antidiabetics. Vila-Carriles et al. [127] found that the substitution of aromatic or lipophilic heterocyclic groups increased the potency of compounds such as glibenclamide by 100–1000 times and that the increased affinity was the result of interactions with two overlapping binding sites that were analogous to BPU. This can lead to new insecticides through the screening of bioassays, and in this study, the compound LY 131215, which does not contain a urea bridge, had the best result concerning insecticidal activity [128].

After the high-performance pharmacophoric screening of the studied compounds, only **M01** presented urea in its molecular structure. **M01** and its derivatives have 19 studies and patents that are mainly related to antibacterial tests, nitric oxide, DNA inhibition, neuroinflammatory treatment and autoimmune diseases, ribonucleotide reductase, tyrosinase inhibitors, and sickle cell anemia treatments [116,117,129,130,131,132,133,134].

Another widely studied compound, **M05**, has 26 studies related mainly to histone deacetylase, urease, metallo-β-lactamase inhibitor FEZ-1, antitumor activity, DNA synthesis, hydroxamic β-glucosyl-phenolic acid inhibitor, ribonucleotide reductase, 5-lipoxygenase inhibitors, and activity against trypanosome [59,135,136,137,138,139,140,141,142,143]. On the other hand, the **M02** compound has only one study analyzing antibacterial activity [144]; in relation to **M03** and **M04** compounds, there are no biological studies; none of our compound cells were tested against insect-growth-inhibiting activity to transform them into a new insecticide after additional tests in vitro and in vivo.

### 2.6. Prediction of Synthetic Accessibility (SA) and Theoretical Synthetic Routes

**Figure 11:**
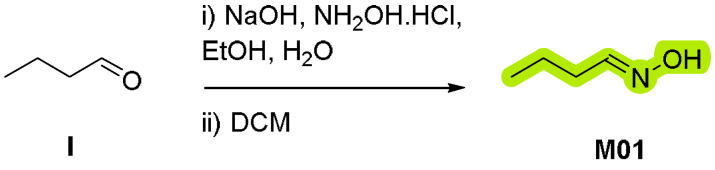
Synthetic route for the preparation of **M01**. DCM (dichloromethane). Starting material **I** is commercially available.

**Figure 12:**
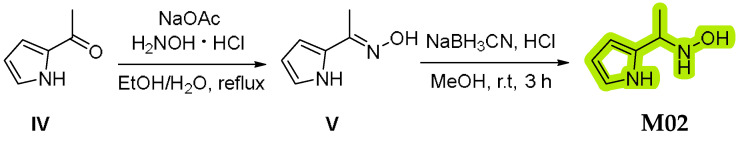
Theoretical synthetic route for the preparation of **M02**. Starting material **IV** is commercially available.

**Figure 13:**
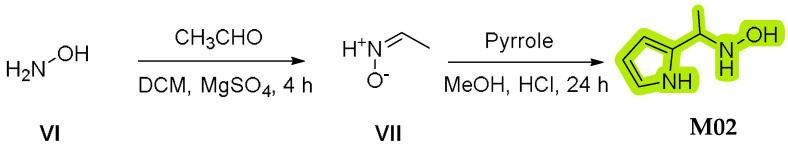
Alternative synthetic route for the preparation of **M02**. Starting material **VI** is commercially available.

**Figure 14:**
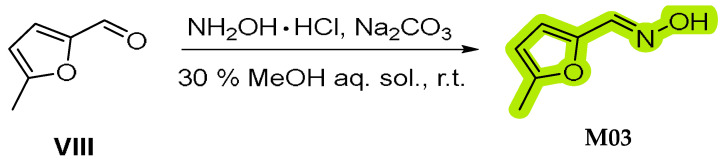
Traditional method for the preparation of compound **M03**. Starting material **VIII** is commercially available.

**Figure 15:**
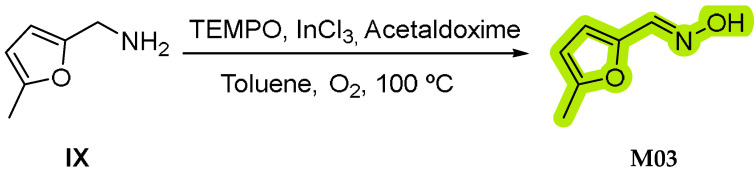
Alternative synthetic route for the preparation of compound **M03**. Starting material **IX** is commercially available.

**Figure 16:**
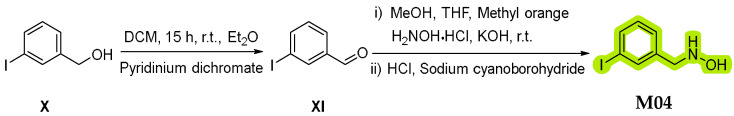
Synthetic route for the preparation of **MO4**. THF (tetrahydrofuran), Potassium hydroxide, Starting material **X** is commercially available.

The selected compounds **M01**–**M05**, after hierarchical virtual screening, were submitted for evaluation of synthetic accessibility (Table 5); all five compounds were easily synthesized, with scores above 89 [145,146,147]. According to classification by score, the value of 100 is the maximum synthetic accessibility; that is, the molecule is more easily synthesized if it has a higher score (see Table 5). In this study, we propose the theoretical synthetic route of promising compounds, as can be seen in Figure 10, Figure 11, Figure 12, Figure 13, Figure 14, Figure 15 and Figure 16.

#### 2.6.1. Theoretical Synthetic Route (Compound **M01**)

Compound M01 can be prepared according to the research carried out by B. Buettelman et al. [148]. Conversion of butyraldehyde **I** with hydroxylamine hydrochloride (NH_2_OH·HCl) in EtOH and H_2_O as solvents and using NaOH as a base will allow us to obtain the final butyraldehyde oxime—**M01** (Figure 11).

#### 2.6.2. Theoretical Synthetic Route (Compound **M02**)

We propose a theoretical synthetic route for **M02** based on the preparation of oxime derivative **V** as an intermediate, with **IV** and NH_2_OH·HCl as initial reactants and NaOAc and EtOH/H_2_O as solvents under reflux conditions [149]. By a reduction reaction with sodium cyanoborohydride (NaBH_3_CN) of intermediate **V**, final hydroxylamine **M02** will be obtained [150] (Figure 12).

Furthermore, we designed a secondary strategy based on the previous work of C. Berini et al. [151]. Firstly, nitrone **VII** could be furnished by condensation between hydroxylamine **VI** and acetaldehyde with magnesium sulfate in DCM. Eventually, the nucleophilic addition of pyrrole to intermediate **VII** using HCl in MeOH would originate the target compound (Figure 13).

#### 2.6.3. Theoretical Synthetic Route (Compound **M03**)

Oxime derivative **M03** can be synthesized by a condensation reaction (Figure 14) between aldehyde **VIII** and hydroxylamine hydrochloride in a 30% MeOH aqueous solution [152].

In order to improve the yield, we suggest the use of a green chemistry procedure by J. Yu et al. [153]. The final compound **M03** will be formed through an aerobic oxidation of amine **IX** with (2,2,6,6-tetramethylpiperidin-1-yl)oxyl (TEMPO), InCl_3_, and acetaldoxime as catalysts, using toluene as a solvent (Figure 15).

#### 2.6.4. Theoretical Synthetic Route (Compound **M04**)

We present a synthetic route to synthesize compound **M04** (Figure 16) based on the formation of iodobenzaldehyde **XI** from iodobenzyl alcohol **X** using pyridinium dichromate as an oxidizing agent and DCM and Et_2_O as solvents. The condensation reaction between intermediate **XI** and hydroxylamine hydrochloride and the subsequent reduction with sodium cyanoborohydride will furnish the desired *N*-(iodobenzyl) hydroxylamine **M04** [154].

#### 2.6.5. Theoretical Synthetic Route (Compound **M05**)

**Figure 17:**
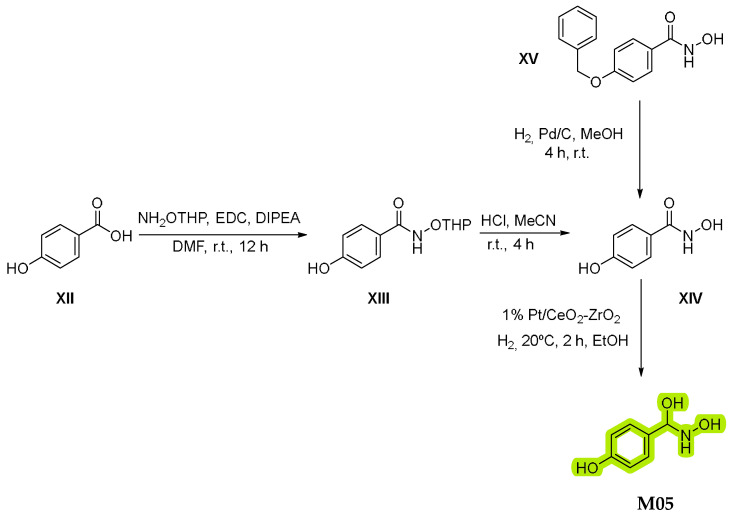
Theoretical synthetic route for the preparation of **M05**. Starting materials **XII** and **XV** are commercially available.

We proffer a theoretical synthetic route for compound **M05** using intermediate **XIV** as the key substrate. The initial strategy to obtain **XIV** consists of two steps [155]. Firstly, the coupling reaction of acid derivative **XII** with *o*-(tetrahydro-2*H*-pyran-2-yl) hydroxylamine (NH_2_OTHP) in DMF with 1-ethyl-3-(3-dimethylaminopropyl) carbodiimide (EDC) and *N,N*-diisopropylethylamine (DIPEA) will provide derivative **XIII.** Then, the cleavage of the THP-protecting group in acidic conditions will produce benzamide **XIV**. The second possibility of synthesizing the key intermediate can be through palladium-catalyzed hydrogenation [156], removing the benzyl group of starting material **XV**. Finally, the target compound **M05** can be furnished from intermediate **XIV** by a hydrogenation reaction using platinum/ceria-zirconia mixed oxide (Pt/CeO_2_-ZrO_2_) as a catalytic system [157] (Figure 17).

## 3. Materials and Methods

### 3.1. Dataset

**Figure 18:**
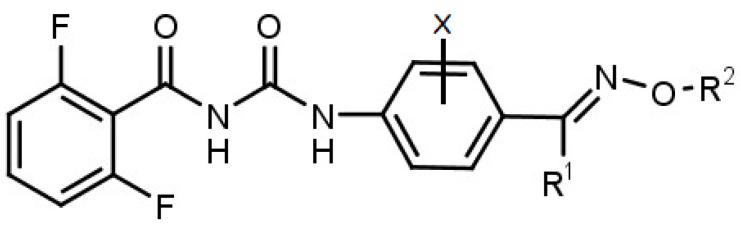
The 2D structure of benzoylphenylurean derivatives. R1 and R2 are radicals, and X = methyl or halogens.

A dataset of 14 benzoylphenylurean analogs was chosen to determine the sets for training and testing as they were designed and synthesized from the reference structure, with modifications at specific points to generate the model from a single base structure (Figure 18); the dataset had experimental data with excellent larvicidal activity [32], potentially for the chitin synthase and the juvenile hormone receptor.

**Figure 19:**
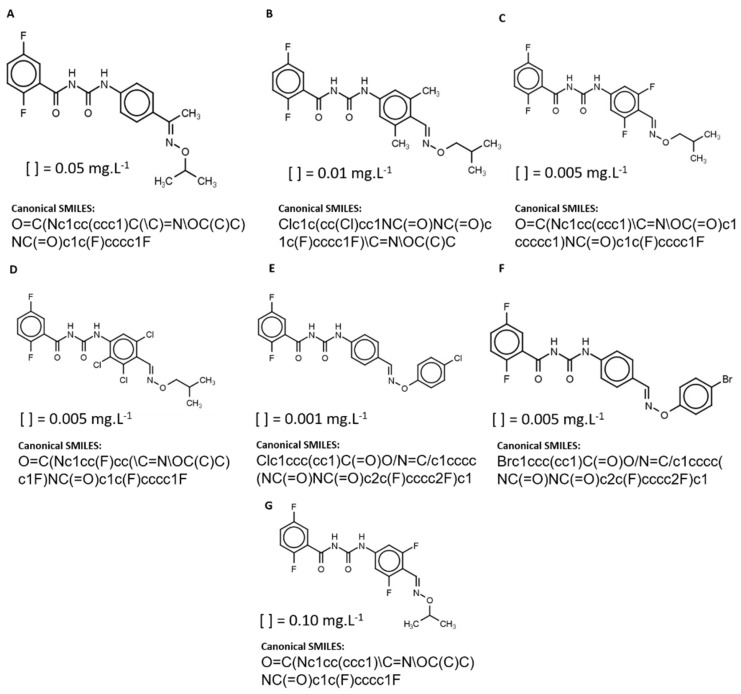
Structures of selected training set compounds, with canonical smiles and concentrations ([ ]) with 100% larvicidal activity (mg·L^−1^). (**A**)—Compound **01**; (**B**)—Compound **05**; (**C**)—Compound **06**; (**D**)—Compound **07**; (**E**)—Compound **12**; (**F**)—Compound **13**; (**G**)—Compound **16**.

GALAHAD^TM^ (Tripos Inc.), was employed to build ten pharmacophore models from a training set (see Figure 19) of previously related inhibition activity to chitin synthase and juvenile hormone receptors [32,120].

First, they were drawn using Marvin^TM^ Sketch 16.9.5 software (CHEMAXON, Budapest, Hungary, https://www.chemaxon.com/, accessed on 15 March 2021), from which we selected the most reliable tautomers (pH = 6.5) for all compounds [158]. Next, 2D structures were converted to a 3D format by CONCORD implemented in the SYBYL-X^TM^ 2.0 package (Tripos Inc.). All structures were energy-minimized using a conjugate gradient (CG) with a convergence criterion of 0.001 kcal/mol and a Tripos force field (dielectric constant ε = 80.0 and maximum number of iterations = 50,000) [159]. Partial atomic charges were calculated using the Gasteiger-Hückel method [160], available on SYBYL-X 2.0^TM^ (Tripos Inc.).

The study of larvicidal activity was carried out by Sun [32] et al., from which the fourteen compounds with 100% larvicidal activity at low concentrations (2 to 0.001 mg·L^−1^) were selected, seven of which were used to determine the training set and seven for the test set. They were used randomly in the construction of the pharmacophore models, while the other compounds with the same criteria were used in the validation of the pharmacophore model.

### 3.2. Pharmacophore Model Generation

The training datasets were flexibly aligned to each other in order to build hypermolecular alignments that map common pharmacophore features. The genetic algorithm employed in this step starts with 45 conformations (population size) of each benzoylphenylurean derivative and evolves through a maximum of 90 generations by means of default genetic operators (mutation rate = 0.2 and crossover rate = 0.2) available in the GALAHAD^TM^ module in SYBYL-X^TM^ 2.0 [161] (Tripos Inc.).

### 3.3. Pharmacophore Model Evaluation

Pharmacophore models with the lowest energy (<100.0 kcal/mol) were selected and analyzed for the results of the Pareto score. To determine the best pharmacophore model and its discriminatory power, the models were used to build decoys that were removed from the DUD-E server, March (http://dude.docking.org/, accessed on 15 March 2021), [162] and executed in the SigmaPlot^TM^ program v. 12.0 (SYSTAT, Leland Wilkinson, Chicago, IL, USA) by calculating the area under the receiver operating characteristics curve (AUC-ROC curve); it was possible to visualize the recognition of assets and decoys for better validation of the models through the true-positive rate versus the false-positive rate.

The Boltzmann-enhanced discrimination of the ROC curve (BEDROC) was employed to discriminate models with AUC > 0.7 according to early recognition of actives using the ROCKER server [163] (www.jyu.fi/rocker, accessed on 15 March 2021). Additionally, BEDROC is oriented by a search algorithm (α = 16.10) that corresponds to the initial 10% of the database. The pharmacophore model that demonstrated early recognition (BEDROC > 0.5) was employed in the virtual screening step.

### 3.4. Pharmacophore-Based Virtual Screening

The best pharmacophore model was automatically converted to the UNITY^TM^ 3D query that was employed to virtually screen the Sigma-Aldrich subset from the ZINC database [34] (https://zinc15.docking.org/, accessed on 15 March 2021). Compounds were ranked according to QFIT values, and those with values higher than mean plus two times standard deviation (Equation (1)) were selected for molecular docking.

X = x + 2×σ(1)

X = QFIT value
x = Average
σ = Standard deviation

Equation (1): Mathematical equation of the mean plus two times the standard deviation.

### 3.5. Homology Modeling, Molecular Docking, and Virtual Screening for Potential Dual Modulators

The primary sequence of *Aedes aegypti* species was collected in the National Center for Biotechnology Information (NCBI), with the descriptives (*Aedes aegypti* + chitin deacetylase 1 protein) at https://www.ncbi.nlm.nih.gov/protein, accessed on 27 June 2021. We found that the protein AAEL003419-PA was used as a primary protein; the BLAST^TM^, https://blast.ncbi.nlm.nih.gov/Blast.cgi, accessed on 27 June 2021 [164] performed and compared the primary biological sequences and found the most similar and statistically significant ones in the PDB (Protein Data Bank).

The most similar macromolecules were introduced and analyzed in the MODELLER 10.1 program, which, with the primary structure of *Aedes aegypti*, performed the comparison, alignment, construction, and validation of the model, called Bombyx-Aedes.

Using the SWISS-MODEL server [165,166,167], structure assessment (https://swissmodel.expasy.org/assess, accessed on 27 June 2021) was employed to analyze the model created for *A. aegypti* chitin deacetylase using the global model quality estimation (GMQE) score on the accuracy of the constructed model and the model used [168]; the prioritized model had a GMQE score of <0.50 [169]. Furthermore, Ramachandran plots were used to evaluate the energetically favored regions of the model [170]. In the Pymol program, the 3D alignment between the structures was performed, which collected the coordinates of the alpha-carbons of each of the residues in this analysis.

The conformational search and scoring evaluation were performed using AutoDock Vina 1.1.2 (Scripps Research Institute, Olson Laboratory, La Jolla, CA, USA). The ability to generate satisfactory solutions to scoring functions was probed according to the root mean square deviation value (RMSD < 2Å) [171,172].

The compounds prioritized in pharmacophore-based virtual screening were employed in molecular docking, and in silico approaches of the *Aedes aegypti* juvenile hormone receptor (PDB: 5V13) were performed according to previous studies [173,174]. After the molecular docking, ligand efficiency (LE) was calculated through the binding affinity of compounds employed in docking studies (Equation (2)), and the compounds with LE < −0.4 kcal/mol were prioritized to dockig with the *A. aegypti* chitin deacetylase receptor model.

LE = −ΔG/N(2)

LE = Ligand efficiency
ΔG = Binding affinity
N = Number of heavy atoms

Equation (2). Mathematical equation of ligand efficiency.

The ligand efficiency (LE) was calculated for compounds employed in docking with the *A. aegypti* chitin deacetylase receptor, and compounds with LE < −0.4 kcal/mol were prioritized for pharmacokinetic and toxicological analysis.

### 3.6. In Silico Pharmacokinetic and Toxicological Properties

For the analysis of pharmacokinetic properties, the best compounds from the previous step were selected, and we used the Preadmet (https://preadmet.bmdrc.kr/, accessed on 27 June 2021) and SwissADME tools [79,175] and the proficient method of cerebral or intestinal permeation Estimation D (BOILED-Egg) [78] (http://www.swissadme.ch/index.php, accessed on 27 June 2021). The analysis of the toxicity profile of the compounds was evaluated using the online platform eMolTox (http://xundrug.cn/moltox, accessed on 27 June 2021).

### 3.7. Synthetic Accessibility (SA) Prediction

The prediction of the synthetic accessibility (SA) of the compounds selected by the hierarchical virtual screening was performed using the AMBIT program (http://ambit.sourceforge.net/reactor.html, accessed on 10 July 2021), which implements the tautomer generations by the algorithm method of combinatorics, exhaustively calculating all tautomeric forms of a given organic compound by molecular and stereochemical complexity and considering the presence of fused and bridged systems to suggest the canonical tautomer by an energy base of classification by different structural and topological characteristics combined with an additive scheme using weighted molecular descriptors. By generating a score ranging from 0 to 100 (maximum synthetic accessibility), the molecule is more easily synthesized when the score is closer to 100 [176,177,178,179].

## 4. Conclusions

This study has established the planning of insect-growth-inhibiting compounds with potential insecticidal activity that have the possible mechanism of action of dual inhibition of the juvenile hormone protein; the study has also established a chitin deacetylase model of *Aedes aegypti* through molecular modeling approaches via pharmacophore and virtual screening based on molecular docking by predicting molecular target binding. In addition, compounds were developed through the pharmacokinetic characteristics (ADME) of the physiology of the *Aedes aegypti* mosquito, and toxicological properties directed at other organisms, mainly humans, as well as the synthetic theoretical routes of compounds **M01**, **M02**, **M03**, **M04**, and **M05** have been proposed.

Finally, compounds **M01**–**M05** were selected, showing parameters similar and superior to those observed for positive controls and also significant differences in the interaction with key residues in each catalytic protein site reported in the literature. Accordingly, the molecules investigated here are dual inhibitors of the enzymes chitin synthase and juvenile hormonal protein (from insects), characterizing them as potential insecticides against the mosquito *Aedes aegypti*. With these promising results, our research group intends to carry out biological tests in vivo with the compounds obtained in this work to confirm the in silico predictions.

## Figures and Tables

**Table 1:** Internal statistical parameters for pharmacophore models provided by GALAHAD™.

Model	ENERGY (kcal/mol)	STERICS	H-BOND	MOL_QRY
01	5.55	2662.60	627.30	365.56
02	6.83	2502.50	669.00	378.08
03	6.62	2724.20	646.60	358.13
04	6.88	2888.40	619.20	356.44
05	6.35	2496.40	650.00	362.48
06	6.73	2876.90	610.40	350.63
07	7.87	2936.50	641.60	357.15
08	6.31	2604.20	647.90	343.24
09	7.03	2638.60	674.00	335.78
10	6.82	2802.00	611.70	346.41

**Table 2:** BEDROC value of pharmacophore models, considering α = 16.10.

Models	BEDROC
01	0.36
02	0.30
04	0.41
05	0.23
06	0.60
07	0.49
09	0.52
10	0.44

**Table 3:** Pharmacokinetic properties of selected molecules.

In Silico Pharmacokinetic Properties					
COD	SwissADME		PREADMET		
	Lipophilicity	Water Solubility			
	Consensus log P_o/w_ ^1^	$\overset{-}{X}$ Log *S*	BBB ^2^	Caco-2 ^3^	MDCK ^4^
DFB	3.32	−5.06	2.25	20.47	0.53
TEF	4.43	−5.94	4.66	23.09	0.07
FCX	5.37	−8.23	2.29	25.12	0.05
BPU	3.35	−4.57	0.13	56.97	2.61
M01	0.55	−1.46	0.53	20.77	247.45
M02	0.90	−1.55	1.70	13.70	31.05
M03	1.46	−2.13	0.61	3.20	395.90
M04	2.42	−3.43	1.61	19.29	0.46
M05	0.28	−1.04	0.35	19.06	3.21

^1^ Log P_o/w_, partition coefficients for octanol/water); ^2^ BBB, in silico prediction of brain/blood partition coefficient; ^3^ Caco-2, in silico prediction of colon adenocarcinoma cell; ^4^ MDCK, in silico prediction of a Madin-Darby canine kidney cell.

**Table 4:** Toxicological properties of selected molecules in the toxicity model—eMolTox.

COD	Action	Injury	Confidence
FCX	Agonist of liver X receptor beta	Liver	0.993
	Antagonist of the farnesoid-X receptor (FXR) signaling pathway		0.995
	Disruptors of the mitochondrial membrane potential		0.981
	Block Bile Salt Export Pump		0.991
	Differential cytotoxicity against isogenic chicken DT40 cell lines with known DNA damage response pathways—Rad54/Ku70 mutant cell line	Genotoxicity	0.992
	Differential cytotoxicity (isogenic chicken DT40 cell lines)	Genotoxicity	0.986
DFB	Activator of the aryl hydrocarbon receptor (AhR) signaling pathway	Liver	0.997
	Modulator of P2X purinoceptor 7	Nervous system, immune, kidney	0.985
TEF	Modulator of P2X purinoceptor 7	Nervous system, immune, kidney	0.993
BUP	Differential cytotoxicity against isogenic chicken DT40 cell lines with known DNA damage response pathways—Rad54/Ku70 mutant cell line	Genotoxicity	0.992
	Antagonist of the estrogen receptor alpha (ER-alpha) signaling pathway	Endocrine	0.996
	Activators of the human pregnane X receptor (PXR) signaling pathway	Liver	0.996
	Agonist of the constitutive androstane receptor (CAR) signaling pathway		0.992
M01	We do not find any toxic action from data-driven models.		
M02	Block OATP1B1 Transporter	Liver	0.993
M03	Block OATP1B1 Transporter	Liver	0.982
M04	Skin Sensitization	Skin	0.999
	Block OATP1B1 Transporter	Liver	0.999
M05	We do not find any toxic action from data-driven models.		

**Table 5:** Prediction of synthetic accessibility (SA).

COD	COMPOUNDS	SA
**M01**	ZINC13514543	96.096
**M02**	ZINC14413017	92.731
**M03**	ZINC05680928	89.776
**M04**	ZINC04691148	92.951
**M05**	ZINC95851150	91.439

## Data Availability

Not applicable.

## Supplementary Materials

The following supporting information can be downloaded at: https://www.mdpi.com/article/10.3390/ijms23158218/s1.
