# Supplementary material for: Identification of Potential Insect Growth Inhibitor against Aedes aegypti: A Bioinformatics Approach
Source: Int J Mol Sci. 2022 Jul 26;23(15):8218. doi: 10.3390/ijms23158218 (PMC9332482; doi:10.3390/ijms23158218)
Supplement: Supplementary file 1 [file ijms-23-08218-s001.zip › ijms-1756683-supplementary.pdf]

International Journal of *Molecular Sciences*

Supplementary Materials

Identification of Potential Insect Growth Inhibitor against *Aedes aegypti*: A Bioinformatics Approach

Glauber V. Da Costa [1,2,3], Moysés F. A. Neto [4], Alicia K. P. Da Silva [3], Ester M. F. De Sá [3], Luanne C. F. Cancela [3], Jeanina S. Vega [3], Cássio M. Lobato [2,3], Juliana P. Zuliani [5], José M. Espejo-Román [6], Joaquín M. Campos [6], Franco H. A. Leite [4] and Cleydson B. R. Santos [1,2,6,*]

[1] Graduate Program in Network in Pharmaceutical Innovation, Federal University of Amapá, Macapá 68902-280, AP, Brazil; vilhenac@hotmail.com
[2] Laboratory of Modeling and Computational Chemistry, Department of Biological and Health Sciences, Federal University of Amapá, Macapá 68902-280, AP, Brazil; cassio153bio@gmail.com
[3] Laboratory of Biotechnology in Natural Products, Department of Biological and Health Sciences, Federal University of Amapá, Macapá 68902-280, AP, Brazil; aliciakarine1@gmail.com (A.K.P.D.S.); estersa07@gmail.com (E.M.F.D.S.); cancelaluanne@gmail.com (L.C.F.C.); js.vega@hotmail.com (J.S.V.)
[4] Laboratory Molecular Modeling, State University of Feira de Santana, Feira de Santana 44036-900, BA, Brazil; moysesfagundes@gmail.com (M.F.A.N.); fhpharm@gmail.com (F.H.A.L.)
[5] Laboratory Cellular Immunology Applied to Health, Oswaldo Cruz Foundation, FIOCRUZ Rondônia, Porto Velho 78912-000, RO, Brazil; juliana.zuliani@fiocruz.br
[6] Department of Pharmaceutical and Organic Chemistry, Faculty of Pharmacy, Institute of Biosanitary Research ibs, University of Granada, 18071 Granada, Spain; josemanuelespejo@correo.ugr.es (J.M.E.-R.); jmcampos@ugr.es (J.M.C.)
* Correspondence: breno@unifap.br; Tel.: +55-096-4009-2699

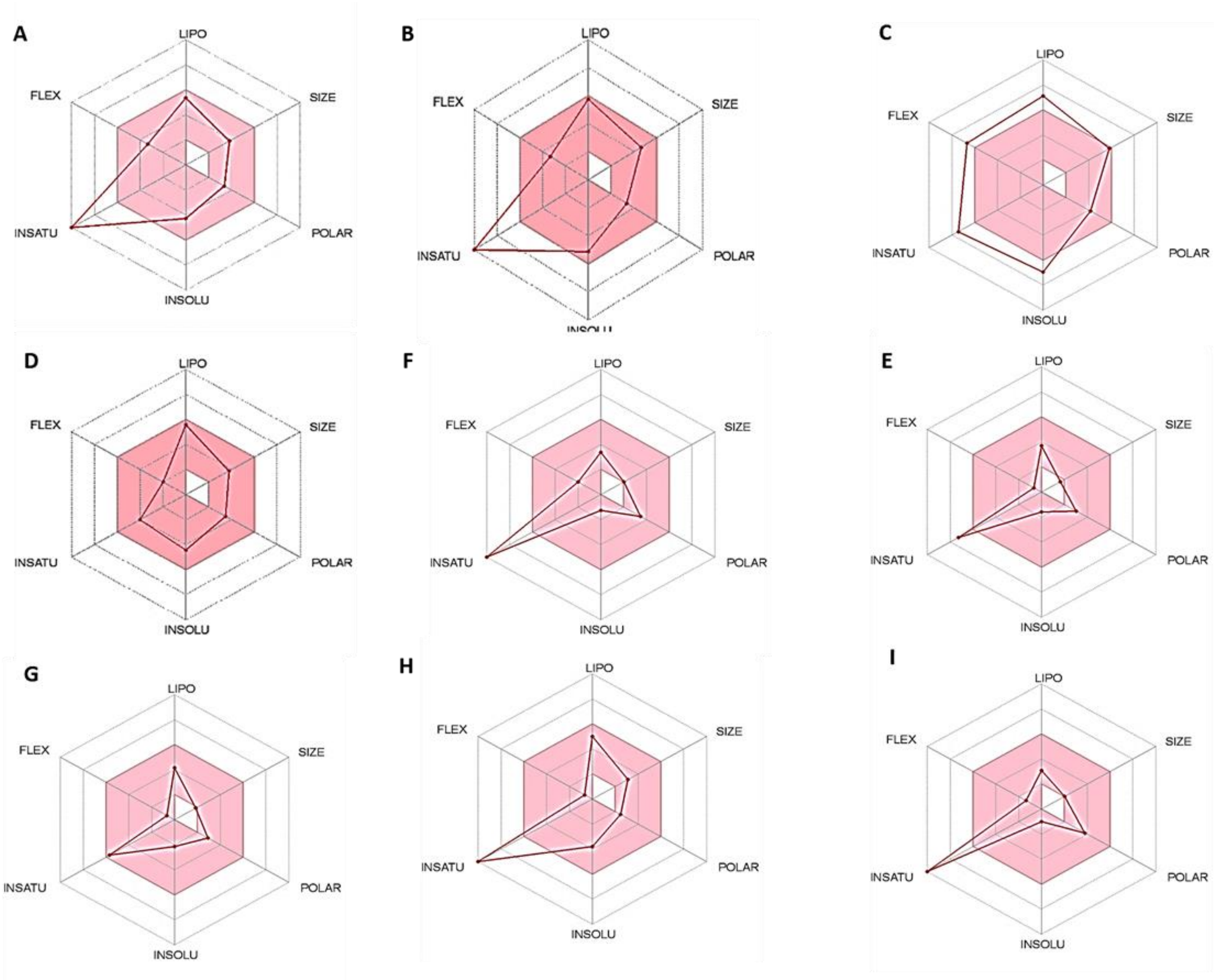

Figure S1. Pharmacokinetic properties by bioavailability radar of positive controls in A - Diflubenzuron ; B - Teflubenzuron; C - Fluxycloxuron; D - Buprofezin; E- (M01) ZINC00161805; F- (M02) ZINC14413017; G - (M03) ZINC05680928; H - (M04) ZINC04691148; I - (M05) ZINC95851150.

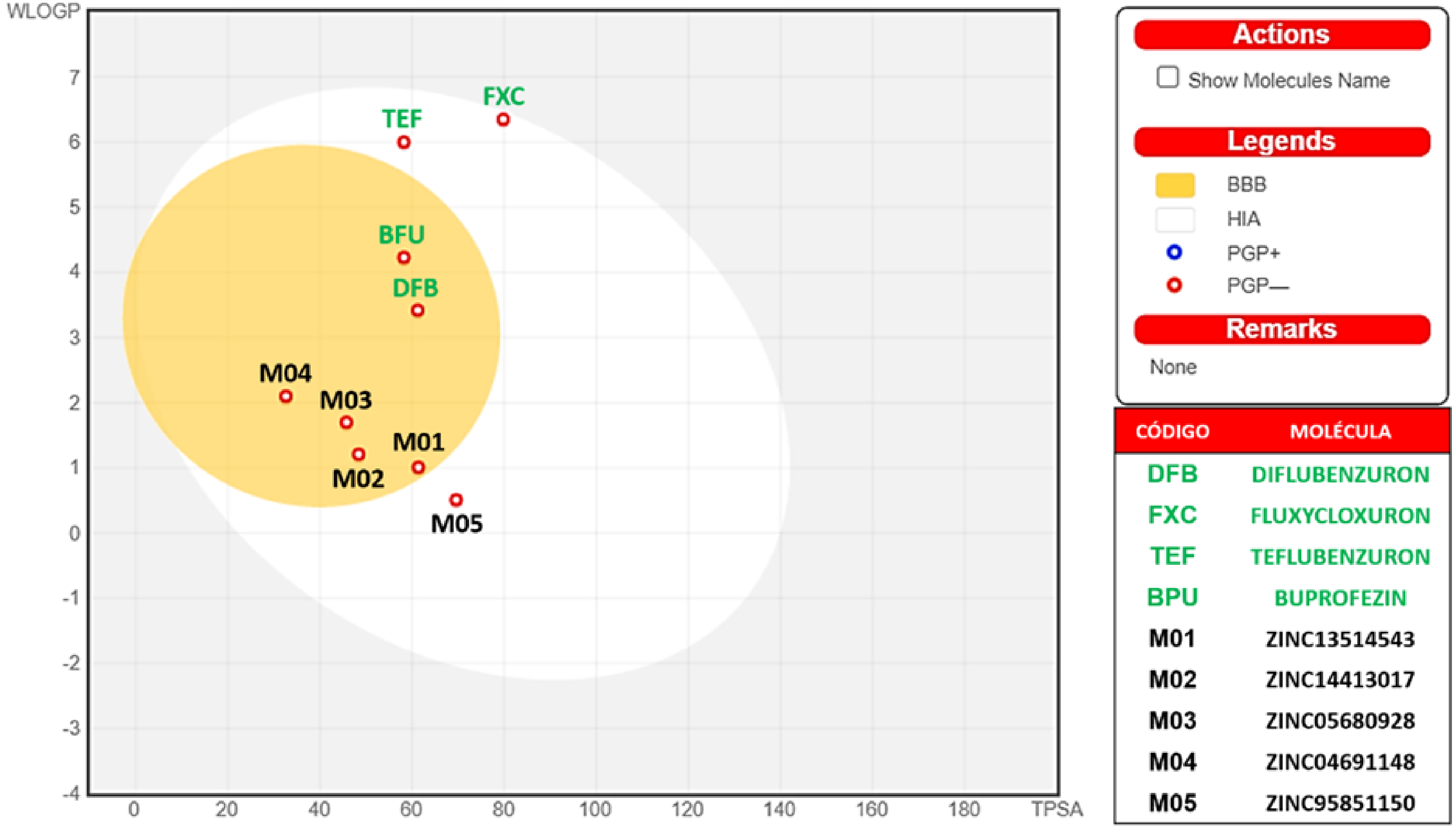

Figure S2. BOILED-EGG depicts gastrointestinal absorption and brain penetration of the compounds and positive controls (DFB – DIFLUBENZURON, FCX – FLUCYCLOXURON, TEF –TEFLUBENZURON, BPU – BUPROFEZIN, M01 – ZINC00161805, M02 – ZINC14413017, M03 –ZINC05680928, M04 – ZINC0491148 and, M05 - ZINC95851150).
